# Soil fungal community characteristics vary with bamboo varieties and soil compartments

**DOI:** 10.3389/fmicb.2023.1120679

**Published:** 2023-02-06

**Authors:** Wen Guo, Jian Zhang, Mai-He Li, Lianghua Qi

**Affiliations:** ^1^Key Laboratory of National Forestry and Grassland Administration/Beijing Bamboo and Rattan Science and Technology, International Centre for Bamboo and Rattan, Beijing, China; ^2^Forest Dynamics, Swiss Federal Institute for Forest, Snow and Landscape Research WSL, Birmensdorf, Switzerland; ^3^Key Laboratory of Geographical Processes and Ecological Security in Changbai Mountains, Ministry of Education, School of Geographical Sciences, Northeast Normal University, Changchun, China; ^4^School of Life Science, Hebei University, Baoding, China; ^5^Sanya Research Base, International Centre for Bamboo and Rattan, Sanya, China

**Keywords:** fungal diversity, network analysis, FUNGuild, *Phyllostachys edulis*, soil compartment, soil fungi, bamboo variety

## Abstract

Soil fungi play an important role in nutrient cycling, mycorrhizal symbiosis, antagonism against pathogens, and organic matter decomposition. However, our knowledge about the community characteristics of soil fungi in relation to bamboo varieties is still limited. Here, we compared the fungal communities in different soil compartments (rhizosphere vs. bulk soil) of moso bamboo (*Phyllostachys edulis*) and its four varieties using ITS high-throughput sequencing technology. The fungal α diversity (Shannon index) in bulk soil was significantly higher than that in rhizosphere soil, but it was not affected by bamboo variety or interactions between the soil compartment and bamboo variety. Soil compartment and bamboo variety together explained 31.74% of the variation in fungal community diversity. Soil compartment and bamboo variety were the key factors affecting the relative abundance of the major fungal taxa at the phylum and genus levels. Soil compartment mainly affected the relative abundance of the dominant fungal phylum, while bamboo variety primarily influenced the dominant fungal genus. Network analysis showed that the fungal network in rhizosphere soil was more complex, stable, and connected than that in bulk soil. A FUNGuild database analysis indicated that both soil compartment and bamboo variety affect fungal functions. Our findings provide new insights into the roles of both soil compartments and plant species (including variety) in shaping soil fungal communities.

## 1. Introduction

Changes in rhizosphere fungal communities can affect plant health and development, while bulk soil, as a resource pool for the rhizosphere soil, has a long-term effect on the rhizosphere fungal assemblage (Bledsoe et al., 2020; Fiore-Donno et al., 2022). Hence, soil compartments (rhizosphere and bulk soil), as defined by Steer and Harris (2000) and Schmidt et al. (2019), may help shape the variation of microbial communities. For instance, fungal diversity and functional guild relative abundance (arbuscular mycorrhizae, soil saprotroph) were reported to be higher in the bulk soil of moso bamboo than in the rhizosphere soil, while the relative abundance of the dominant fungal taxa was lower (Li S. et al., 2022). However, other studies indicated that the diversity of fungal communities in rhizosphere soil was higher than in bulk soil while the complexity of the network was lower (Schmidt et al., 2019; Ye et al., 2021). This difference may be caused by differences in the quality and quantity of organic matter substrate, nutrient availability, and the amount of root secretions (Waldrop et al., 2006; Zhang et al., 2017).

The species and even the variety of plants can influence the formation of soil fungal communities, and different plants can exude different qualities and quantities of compounds through the root system, thus causing differences in the diversity and composition of rhizosphere fungal communities (Gomes et al., 2003; Mouhamadou et al., 2013). Moreover, some fungi can indirectly influence the composition of microbial communities by altering the physiology of the host plant or the pattern of root exudation (Söderberg et al., 2002; Gomes et al., 2003). Different plant varieties therefore may lead to variation in fungal community characteristics. For example, differences were observed in the diversity, composition and symbiotic network of soil fungal communities among different varieties of *Zea mays* in China (Kong et al., 2020; Gil-Martínez et al., 2021). However, Li et al. (2018) found that rice variety did not significantly affect fungal abundance or community composition. It is crucial to explore the effects of plant host specificity on fungal communities in order to distinguish between the effects of plants and of soil characteristics.

As the most important economic bamboo varieties, moso bamboo (*Phyllostachys edulis*) accounts for 73% of China’s total bamboo forest area. It is characterized by rapid growth, a thick litter layer, and a well-developed whip-root system (Dixon and Gibson, 2014; Ramakrishnan et al., 2020). Its unique biological properties can influence the characteristics of the soil fungal community, thereby affecting soil nutrient cycling (Fang et al., 2022). To our knowledge, few studies have been conducted to simultaneously investigate fungal community characteristics in relation to both bamboo variety and soil compartment. Differences in root exudates and soil microenvironment due to the large phenotypic variation may affect soil fungal communities and host selection. Here, we sequenced the fungal ITS regions in the bulk soil and rhizosphere soil compartments under moso bamboo and its four varieties to test our hypotheses that (I) the characteristics of soil fungal communities in rhizosphere soil are significantly different from those in bulk soil, and (II) the characteristics of soil fungal communities differ among bamboo varieties.

## 2. Materials and methods

### 2.1. Study site and experimental design

In August 2018, rhizosphere and bulk soils of five moso bamboo varieties were collected from a bamboo germplasm garden in Taiping, Anhui, China, which was planted 10 years earlier. The study area is located at the northern edge of the subtropics, at an elevation of 250 m, and the average annual temperature and precipitation are 15.8°C and 1,560 mm, respectively. The soil type is yellow-red, with a pH of 4.55. The following moso bamboo varieties were selected: PE (*P. edulis*), FT (*P. edulis* f. *tao kiang*), FL (*P. edulis* f. *luteosulcata*), FP (*P. edulis* f. *pachyloen*), and FG (*P. edulis* f. *gracilis*). The size of the sample plots was 20 m × 20 m, and the plots were separated by concrete walls. The soil physical and chemical properties and plant morphological characteristics of the sample plots were provided by Guo et al. (2022). The rhizosphere and bulk soil samples were collected according to the method of Semenov et al. (2020). In this study, there were 30 samples (5 bamboo varieties × 3 replicates × 2 soil compartments), and each soil sample was a composite of five randomly collected subsamples. Visible debris was removed from the samples, which were then sieved, packed in sterile bags, transported to the laboratory at low temperature, and stored in a refrigerator at −80°C for fungal extraction and sequencing analysis.

### 2.2. Fungal community analysis

Genomic DNA extraction was performed using the Power Soil DNA Isolation Kit (MOBIO Laboratories, Carlsbad, CA, United States). The fungal gene region was amplified using the fungal primers ITS3 (5′-GCATCGATGAAGAACGCAGC-3′) and ITS4 (5′-TCCTCCGCTTATTGATATGC-3′) with barcodes (Zhang et al., 2017; Engelhardt et al., 2018; Perez-Mon et al., 2022). The polymerase chain reaction was as follows: 2× Premix Taq (25 μL) + primer-F (1 μL) and primer-R (1 μL) + DNA (3 μL, 20 ng/μL) + nuclease-free water (20 μL). The polymerase chain reaction and purification operations were performed according to the method of Zheng et al. (2021). The PCR reactions contained 25 μL of 2× Premix Taq, 1 μL of each primer, 3 μL DNA (20 ng/μL), and 20 μL ddH_2_O. The thermal cycling conditions of fungi were as follows: 5 min at 94°C, 30 cycles of 30 s at 94°C, 30 s at 52°C, 30 s at 72°C, and 10 min at 72°C.

### 2.3. Bioinformatics analysis

The raw sequences were quality filtered using the QIIME2 pipeline. The forward and reverse reads were merged using PEAR software (version 0.9.8; Gdanetz et al., 2017). Sequences were removed if their mean quality score was <20 or if their length was <200 bp, and ambiguous sequences were also removed. Illumina amplified sequence data were detected and corrected using the DADA2 denoising algorithm, and random resampling was performed at a constant depth of 8,000 sequences per sample. Fungal OTUs were taxonomically identified using the UNITE 8.0 database (Tedersoo et al., 2018). Before alpha (α) analysis, the sequences were normalized according to the lowest number of sequences for a single sample. The sequence files were submitted to the NCBI Sequence Read Archive (SRA) under BioSample accession number SUB12473231.

### 2.4. Statistics analysis

The α diversity (Shannon index) of the fungal communities was calculated using the vegan package (v2.5-7; Oksanen et al., 2020) in the R environment (v4.1.2; R Core Team, 2020). Differences between bamboo varieties and soil compartments were assessed using two-way analysis of variance (ANOVA) using the R package rcompanion (v2.4.15; Fox and Weisberg, 2018). Based on a Bray–Curtis distance matrix, a principal coordinate analysis (PCoA) of the fungal communities was conducted using the vegan package. Permutation multivariate analysis of variance (PERMANOVA) was used to test whether the fungal communities were affected by soil compartment and bamboo variety (*p* < 0.05) at the operational taxonomic unit (OTU) level. The statistical visualization packages ggplot2 (v3.2.1) and ggpubr (v0.4.0) in R were used as part of the above analyses. The relative abundances of major fungal taxa (phyla and genera) were visualized using the R packages statnet (v2019.6) and circlize (v0.4.15), and the effects of soil compartment and bamboo variety on major fungal taxa (phyla and genera) were assessed using two-way ANOVA. Moreover, the FUNGuild database was used to predict fungal function based on relative abundance at the OTU level (Nguyen et al., 2016). This step was performed on the FUNGuild website.^1^ According to the results of the confidence assessment of the FUNGuild database, only the confidence levels “highly probable” and “probable” were used for subsequent analysis. The heat map of fungal functions was visualized using the pheatmap (v1.0.12) R package. The effects of soil compartment and bamboo variety on fungal functions (trophic modes and functional guilds) were assessed using two-way ANOVA.

Fungal networks were constructed for both the rhizosphere and bulk soil. The complexity of the fungal community was evaluated using network analysis, and potential key taxa in the two soil compartments were identified. To reduce the complexity of the networks, only genera with a relative abundance > 0.1% were selected for the analysis. The R packages psych and igraph were used for Spearman’s correlation analysis to construct the fungal co-occurrence networks. To ensure network robustness, only networks with a Spearman’s correlation coefficient of r > 0.80 or r < −0.80 and a corrected *p* < 0.01 were retained (Benjamini and Hochberg false discovery rate, FDR; Langfelder and Horvath, 2012; Gao et al., 2019). The nodes and edges in the constructed networks represented genera and the correlations between pairs of genera, respectively. Following the method by Hough et al. (2020), keystone species were identified based on PageRank scores that quantify connectivity between nodes. The R packages psych and igraph and the software Gephi (v0.9.0; Mendes et al., 2014) were used to calculate node correlations and for network visualization (Bastian et al., 2009).

## 3. Results

### 3.1. Diversity and composition of the soil fungal community

**Figure 1:**
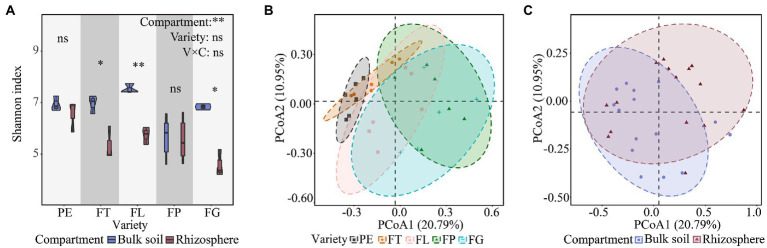
Effects of soil compartment, bamboo variety, and their interactions on fungal α **(A)** and β **(B,C)** diversity. “*”*p* < 0.05, “**”*p* < 0.01. PE, *Phyllostachys edulis*; FT, *P. edulis* f. *tao kiang*; FL, *P. edulis* f. *luteosulcata*; FP, *P. edulis* f. *pachyloen*; FG, *P. edulis* f. *gracilis*.

Soil compartment had a significant effect on the Shannon index of the fungal community (*p* < 0.05), whereas bamboo variety and the compartment × variety interaction effects were not significant (Figure 1A). The Shannon index of the fungal community in the bulk soil was higher than that in the rhizosphere soil for all bamboo varieties. For rhizosphere soil, the Shannon indices of the fungal communities under the five bamboo varieties decreased in the order PE > FL > FP > FT > FG. For bulk soil, the Shannon index was highest under variety FL and lowest under FP (Figure 1A).

The first two axes in the PCoA accounted for 31.74% of the variance in the data matrix, with PCoA1 and PCoA2 representing 20.79% and 10.95%, respectively (Figures 1B,C). PERMANOVA indicated that there was a significant separation of fungal communities between the different soil compartments and bamboo varieties, which differed significantly in distance (*p* < 0.05, Supplementary Table S1). Further, there was an interaction effect between soil compartment and bamboo variety (*p* < 0.05, Supplementary Table S1), and rhizosphere fungal samples showed the highest degree of dispersion.

**Figure 2:**
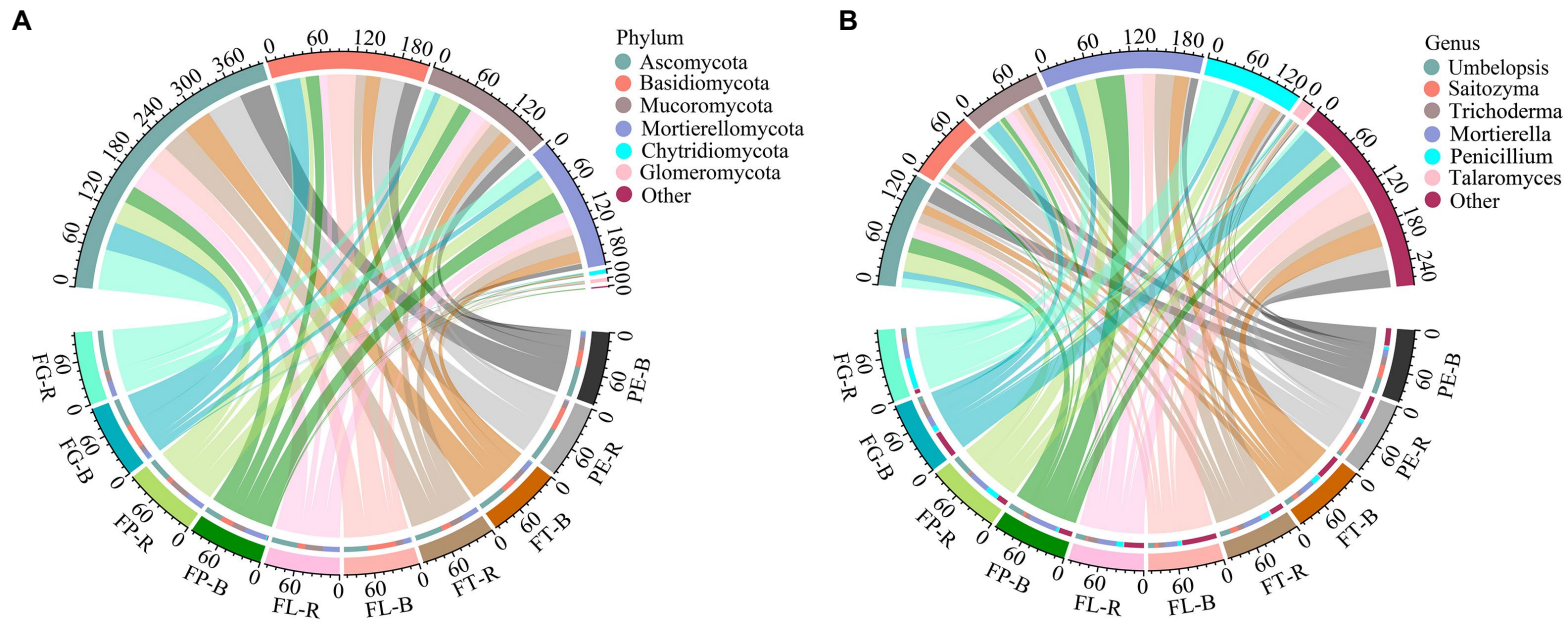
Composition of major fungal taxa at the phylum **(A)** and genus **(B)** level. Rhizosphere soil (R): PE-R, *Phyllostachys edulis*; FT-R, *P. edulis* f. *tao kiang*; FL-R, *P. edulis* f. *luteosulcata*; FP-R, *P. edulis* f. *pachyloen;* FG-R, *P. edulis* f. *gracilis*; Bulk soil (B): PE-B, *P. edulis*; FT-B, *P. edulis* f. *tao kiang;* FL-B, *P. edulis* f. *luteosulcata*; FP-B, *P. edulis* f. *pachyloen;* FG-B, *P. edulis* f. *gracilis*. The first six dominant fungal phyla (genera) with a relative abundance > 1% are shown in figure.

**Figure 3:**
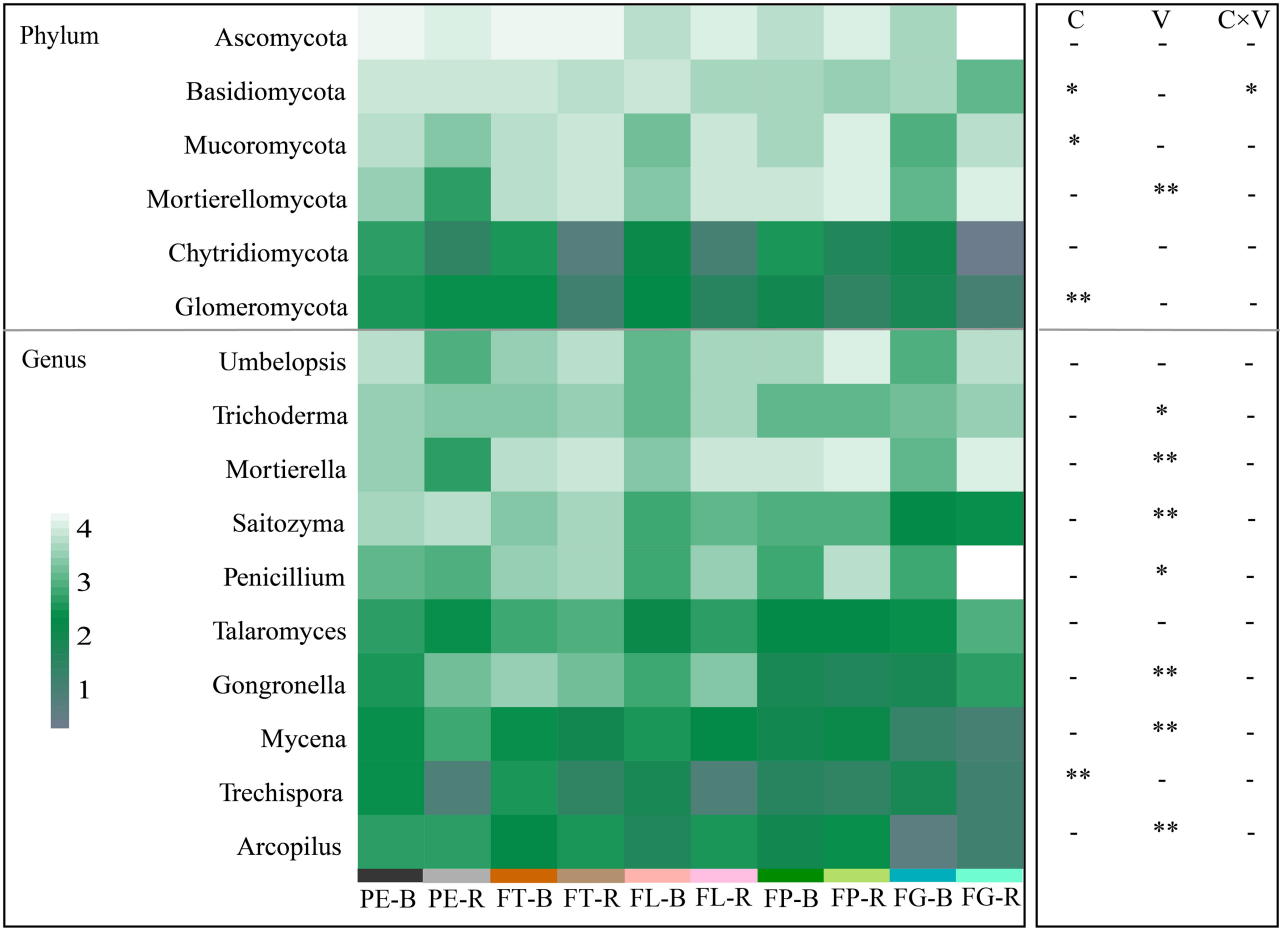
Effects of soil compartment, bamboo variety, and their interactions on the major fungal phyla (top 6) and genera (top 10). C, soil compartment; V, bamboo variety. “*” *p* < 0.05, “**” *p* < 0.01. Rhizosphere soil (R): PE-R, *Phyllostachys edulis*; FT-R, *P. edulis* f. *tao kiang*; FL-R, *P. edulis* f. *luteosulcata;* FP-R, *P. edulis* f. *pachyloen;* FG-R, *P. edulis* f. *gracilis*. Bulk soil (B): PE-B, *P. edulis;* FT-B, *P. edulis* f. *tao kiang;* FL-B, *P. edulis* f. *luteosulcata;* FP-B, *P. edulis* f. *pachyloen*; FG-B, *P. edulis* f. *gracilis*.

The dominant fungal phyla detected included Ascomycota (53.99%–21.74%), Basidiomycota (48.96%–2.39%), and Mortierellomycota (45.69%–1.82%; Figure 2A; Supplementary Table S2). The dominant fungal genera detected included *Mortierella* (53.01%–2.83%), *Umbelopsis* (23.74%–5.11%), and *Trichoderma* (24.21%–5.61%; Figure 2B; Supplementary Table S3). At the phylum level, the effect of soil compartment on the relative abundance of the dominant fungal taxa was stronger than that of bamboo variety. However, bamboo variety significantly affected the relative abundance of dominant fungi at the genus level. Except for Basidiomycota, there was no significant interaction between soil compartment and bamboo variety for the dominant fungal taxa (Figure 3; Supplementary Table S4).

### 3.2. Co-occurrence networks and potential keystone species of the soil fungal communities

**Figure 4:**
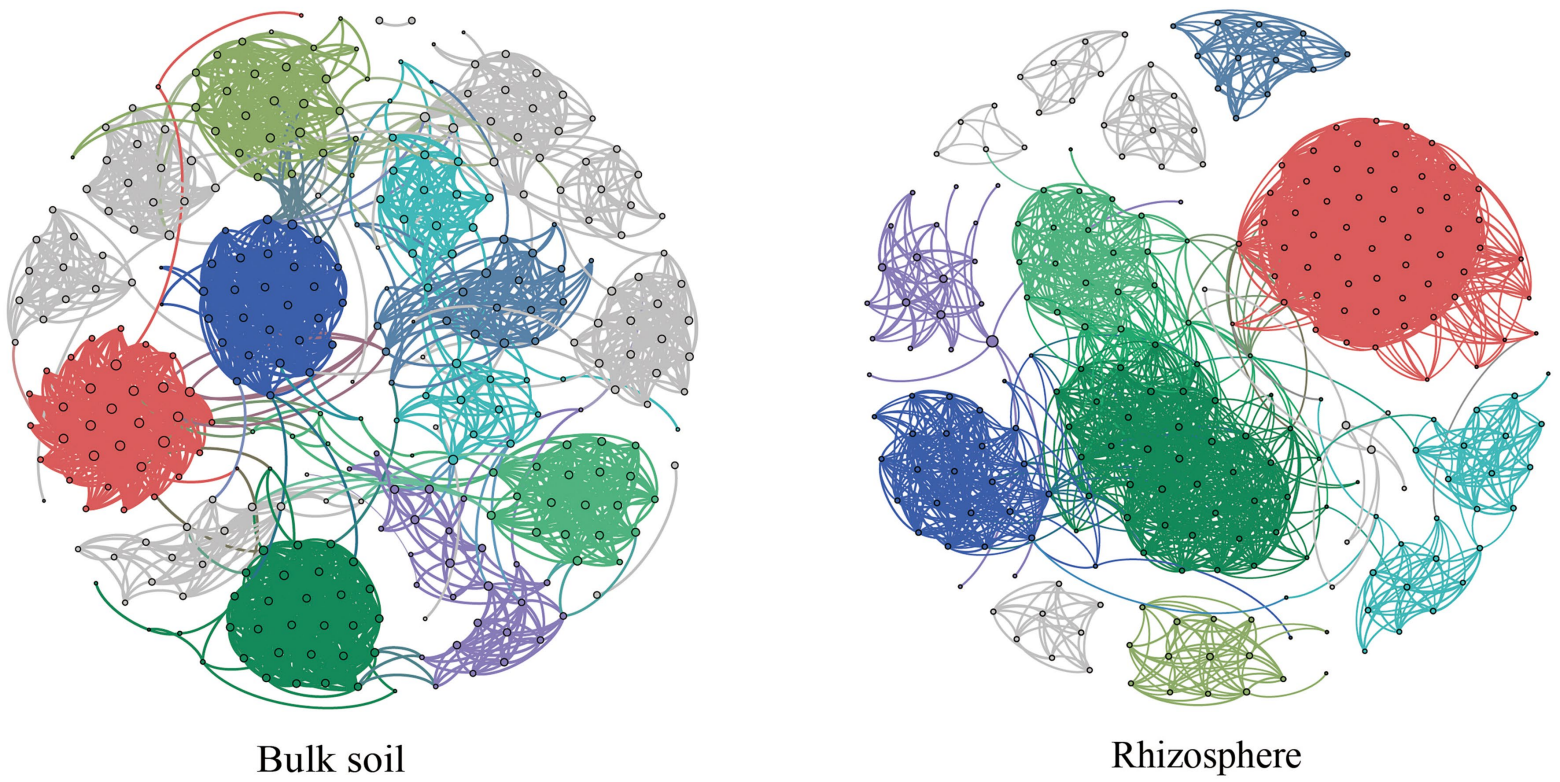
Effect of soil compartment on the co-occurrence network of fungal communities. Nodes and edges indicate genera and the strength of connections between genera, respectively, and different modules are represented by different colors.

Overall, rhizosphere soil (average degree, 20.7) had larger, more connected, and more complex fungal networks than bulk soil (average degree, 14.1), and the fungal communities of the different soil compartments were dominated by collaborative relationships (Figure 4; Supplementary Table S5). Moreover, PageRank screening indicated two keystone species in the rhizosphere (*Simplicillium*, *Nectria*) and in the bulk soil (*Penicillium*, *Laetisaria*; Supplementary Table S6).

### 3.3. Fungal functional guild in relation to soil compartment and bamboo variety

**Figure 5:**
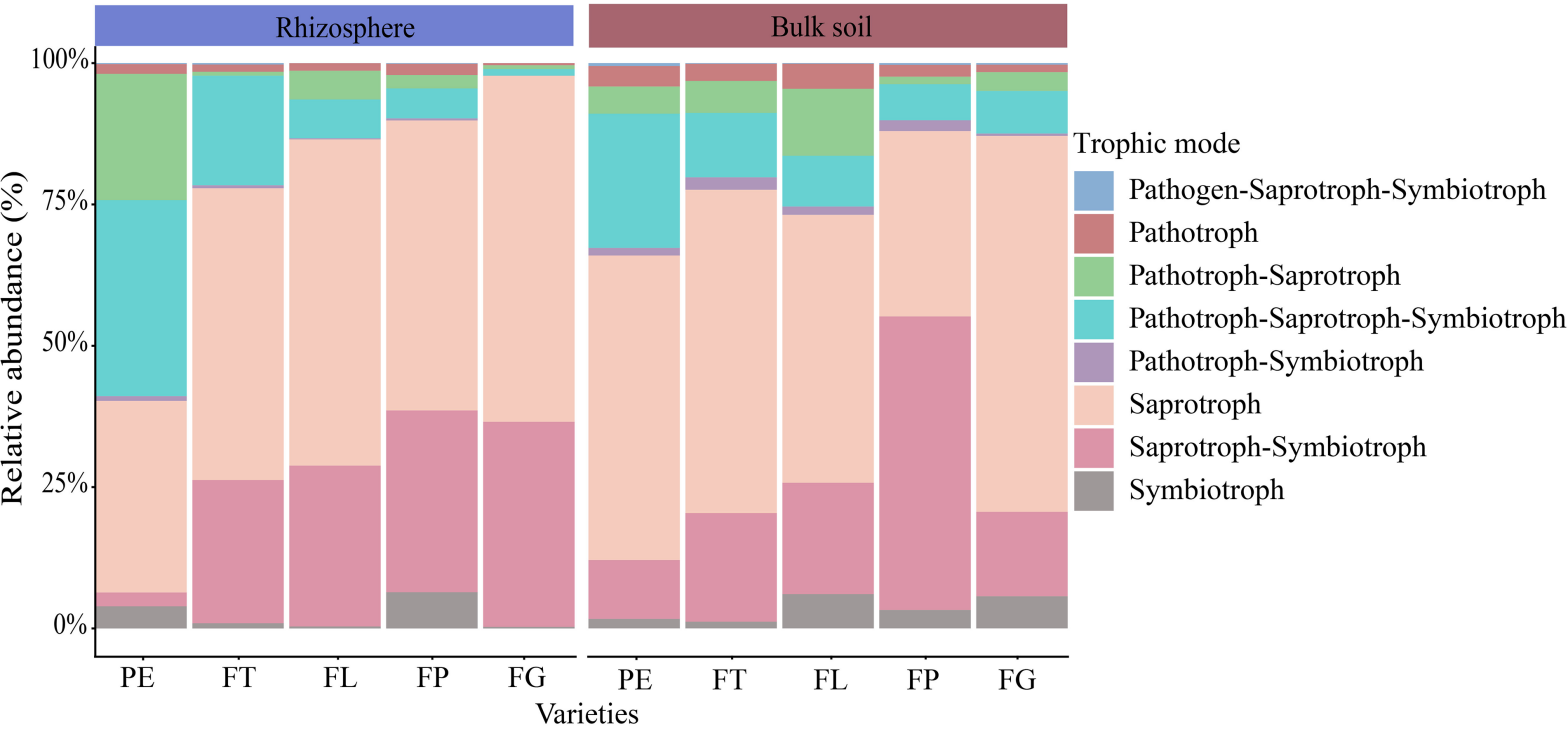
Relative abundance of fungal trophic modes in different soil compartments and bamboo varieties. PE, *Phyllostachys edulis*; FT, *P. edulis* f. *tao kiang*; FL, *P. edulis* f. *luteosulcata*; FP, *P. edulis* f. *pachyloen;* FG, *P. edulis* f. *gracilis*.

**Figure 6:**
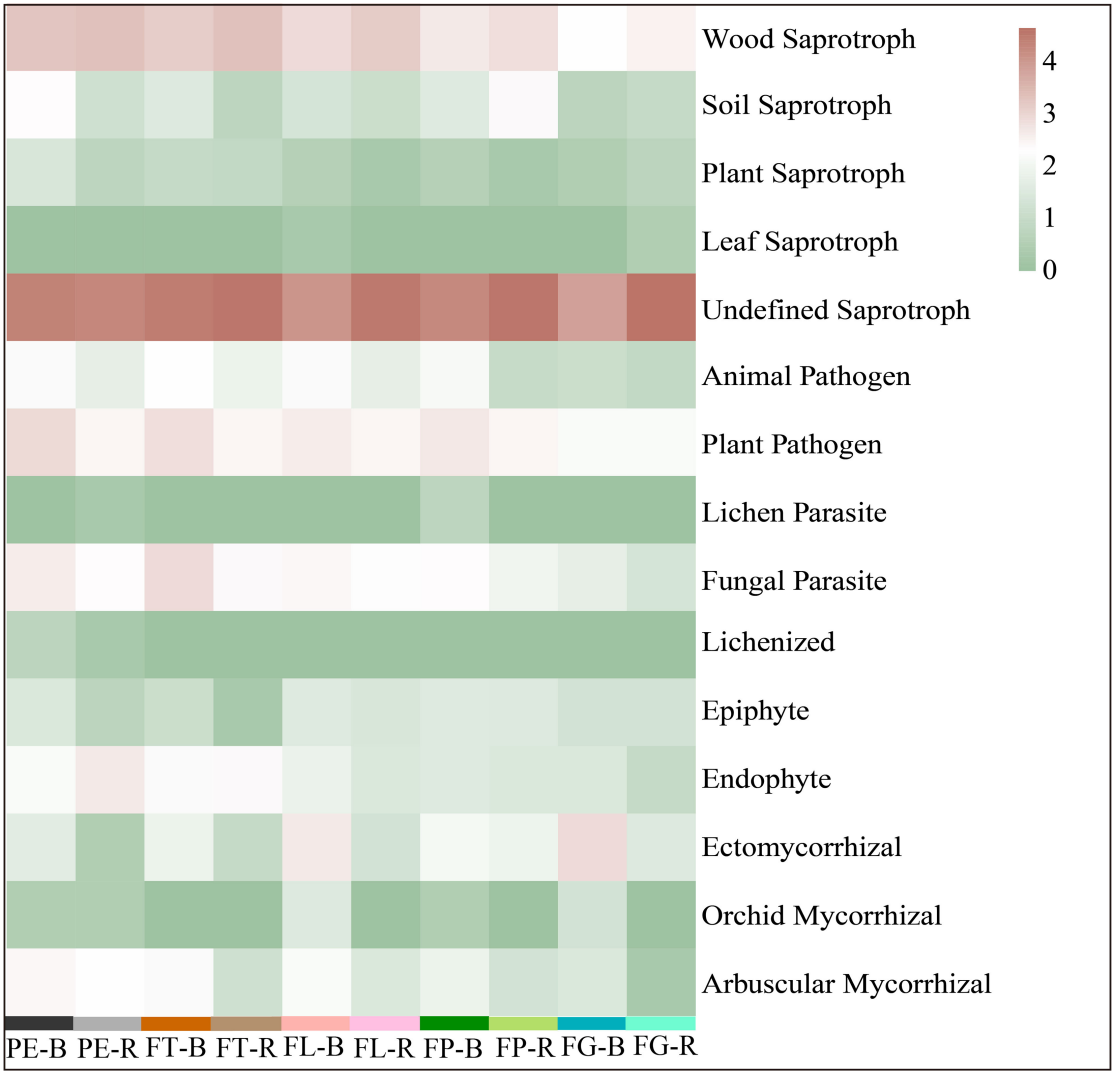
Prediction of the fungal functional guild based on the FUNGuild database. Rhizosphere soil (R): PE-R, *Phyllostachys edulis*; FT-R, *P. edulis* f. *tao kiang*; FL-R, *P. edulis* f. *luteosulcata*; FP-R, *P. edulis* f. *pachyloen*; FG-R, *P. edulis* f. *gracilis*. Bulk soil (B): PE-B, *P. edulis*; FT-B, *P. edulis* f. *tao kiang;* FL-B, *P. edulis* f. *luteosulcata;* FP-B, *P. edulis* f. *pachyloen*; FG-B, *P. edulis* f. *gracilis*.

The trophic mode of the soil fungi under the different varieties of moso bamboo was mainly saprotroph (51%), saprotroph-symbiotroph (24%), pathotroph-saprotroph-symbiotroph (13%), or pathotroph-saprotroph (6%), while the relative abundance of other trophic modes was <5% (Figure 5). Soil compartment significantly affected the relative abundances of the trophic modes pathogen-saprotroph-symbiotroph, pathotroph, pathotroph-symbiotroph, and symbiotroph, and bamboo variety significantly affected pathotroph-saprotroph and pathotroph-saprotroph-symbiotroph, but the interaction between compartment and variety was not significant. Bamboo variety and soil compartment both significantly affected the relative abundances of different fungal functional guilds, but their interaction was not significant (Supplementary Tables S7, S8; Supplementary Figure S1). Moreover, undefined saprotrophs, followed by wood saprotrophs, had the highest relative abundance (Figure 6).

## 4. Discussion

### 4.1. Effects of soil compartment on the characteristics of soil fungal communities

Consistent with our first hypothesis, the diversity of the fungal community differed significantly between the two soil compartments (Figure 1). In this study, the α diversity of fungi decreased from bulk soil to rhizosphere soil, suggesting allelopathic effects of bamboo root exudates on soil fungi. The fungal community associated with the rhizosphere may therefore be influenced by the host, leading to divergent fungal community characteristics in the different soil compartments (Figure 1A), as supported by findings from other recent studies (Urbina et al., 2018; Qin et al., 2022). However, Kong et al. (2020) found that the fungal α diversity in the rhizosphere soil of various maize varieties was higher than that in bulk soil, which could be related to factors such as species specificity and soil substrate (root exudates, cell debris; Nannipieri et al., 2007; Broeckling et al., 2008). In our study, soil compartment additionally significantly affected the relative abundance of fungal phyla (*Basidiomycota*, *Mucoromycota*, *Glomeromycota*) and genera (*Trechispora*; Figure 3; Supplementary Table S4), which may be due to the differentiation of fungal ecological niches across soil compartments. Findings from previous studies support these results (Schöps et al., 2018; Veach et al., 2019). For instance, ectomycorrhizal fungi of the genus *Trechispora* can supply water and nutrients to plant hosts, improve plant resistance to pathogens, and promote seedling growth and development, which may contribute to differences between soil compartments (Van der Heijden et al., 2015; Li Y. et al., 2022).

Also in line with our first hypothesis, the fungal network of the rhizosphere was significantly more complex and connected than that of bulk soil, implying that bulk soil may be more susceptible to external environmental disturbances (Figure 4; Supplementary Table S5). However, Li et al. (2021) reported opposite results to those from our study. It is worth noting that about 20% of plant photosynthesis products can be transported to the soil around the roots through exudation patterns, and the availability of resources to the fungal community, as well as the ecological niche, may alter their interactions, leading to different results (Nehls et al., 2016; Vishwakarma et al., 2017; Fan et al., 2018).

In our study, the keystone species of the rhizosphere and bulk soil were clearly different (Supplementary Table S6). *Simplicillium* and *Nectria* were the keystone taxa in bulk soil, and *Penicillium* and *Laetisaria* in the rhizosphere. Similar findings were reported in previous studies (Su et al., 2020; Liu L. et al., 2021; Varsadiya et al., 2021; Zheng et al., 2021). For instance, *Penicillium* plays a key role in rhizosphere soil, it can generate products that promote plant health (e.g., soluble phosphorus, iron carriers, plant hormones) and affect plant adaptability through a series of biochemical processes (Altaf et al., 2018; Park et al., 2020). Further, *Simplicillium* has a wide range of hosts and substrates, which are associated with bioactive compounds and phytopathogens, and long-term continuous cultivation increases the relative abundance of *Simplicillium* (Wei et al., 2019; Liu Q. et al., 2021). Moreover, keystone species have been found to be involved in synergistic relationships, to change the community structure and function by altering the abundance of synergistic fungi, and even to affect plant growth and its productivity (Van Der Heijden et al., 1998; Banerjee et al., 2018). In this study, soil compartment significantly affected fungal functional diversity, supporting findings from a previous study (Liu et al., 2019). Interestingly, there were differences in resource availability and competition intensity between soil compartments. Fungi can flexibly adapt to changes in microenvironmental conditions, for example by transferring resources to restricted areas, which may be one of the reasons for the functional differences observed between soil compartments (Strickland and Rousk, 2010; Schöps et al., 2018).

### 4.2. Effects of bamboo variety on soil fungal community characteristics

In support of our second hypothesis, our results indicate that bamboo variety significantly affects fungal community β diversity (Figures 1, 3) but not α diversity. A previous study demonstrated that different *Zea mays* varieties had no significant effect on the α diversity of soil fungal communities, but that variety did affect their β diversity to some extent (Kong et al., 2020). Moreover, in our study bamboo variety significantly affected the relative abundance of major fungal genera with important functions (*Trichoderma*, *Mortierella*, *Saitozyma*, *Penicillium*, *Gongronella*, *Mycena*, *Arcopilus*; Supplementary Table S4). For instance, *Trichoderma*, which can promote root growth, regulate nutrient supply, and improve plant health, has been reported to be more efficient and competitive than other soil fungi (Harman et al., 2004; Vinale et al., 2008). Consistent with our findings, different *Solanum tuberosum* varieties have previously been observed to significantly affect fungal community composition (Hannula et al., 2010; Kong et al., 2020). Further, root secretions contain carbon substrates used by fungi, such as primary and secondary metabolites, and different plants can therefore maintain certain resident soil fungal taxa by mediating root secretions (Jones et al., 2004; Broeckling et al., 2008). There were differences in the effects of bamboo variety on the fungal communities, which may be caused by variety-dependent root secretions. Previous studies have indicated that different plant species can maintain resident soil fungal taxa through the mediation of root secretions (e.g., primary and secondary metabolites; Jones et al., 2004; Broeckling et al., 2008).

Our study supports the view that bamboo variety influences the function of soil fungi (Figure 5; Supplementary Table S7). Earlier studies have also shown that fungi are plant-dependent and that plant variety can influence the function of fungi (Berg and Smalla, 2009). However, the extent of this influence also depends, e.g., on the distance of the fungi from the root system and the morphology, biomass, and age of the roots (Marschner et al., 2004; Zhang et al., 2017). For instance, thinner roots may release more readily degradable carbohydrates than coarser roots (Kuzyakov et al., 2000), enhancing interactions with saprophytic fungi in the rhizosphere. This in turn may alter the proportion of strict symbionts and free-living saprotrophic fungi (Lozano et al., 2021), leading to changes in the functioning of the fungi in the community.

In conclusion, in the present study soil fungal community characteristics were significantly affected by both soil compartment and bamboo variety, in line with our two hypotheses. These results may be associated with the amount, chemical properties, and function of the root exudates and soil microenvironment. Our study emphasizes the important roles of soil compartment and plant species, including variety, in shaping soil fungal communities.

## Data availability statement

The data presented in the study are deposited in the NCBI repository, accession number PRJNA771417.

## Author contributions

WG and LQ conceived the experimental design. WG and JZ contributed to the field and indoor experiments. WG and M-HL contributed to the data analysis and manuscript writing. All authors contributed to the article and approved the submitted version.

## Funding

This work was supported by the Fundamental Research Funds for the International Centre for Bamboo and Rattan (1632019010) and the Chinese Scholarship Council (grant no. 202003270039). Open access funding provided by WSL - Swiss Federal Institute For Forest, Snow and Landscape Research.

## Conflict of interest

The authors declare that the research was conducted in the absence of any commercial or financial relationships that could be construed as a potential conflict of interest.

## Publisher’s note

All claims expressed in this article are solely those of the authors and do not necessarily represent those of their affiliated organizations, or those of the publisher, the editors and the reviewers. Any product that may be evaluated in this article, or claim that may be made by its manufacturer, is not guaranteed or endorsed by the publisher.

## Footnotes

^1^ https://github.com/UMNFuN/FUNGuild

## References

[ref1] Altaf M. M. Imran M. Abulreesh H. H. Khan M. S. Ahmad I. (2018). “Diversity and applications of Penicillium spp. in plant-growth promotion,” in New and future developments in microbial biotechnology and bioengineering. eds. Vijai K. G. Susana R. C. (Netherlands: Elsevier) 261–276.

[ref2] Banerjee S. Schlaeppi K. Van Der Heijden M. G. (2018). Keystone taxa as drivers of microbiome structure and functioning. Nat. Rev. Microbiol. 16, 567–576. doi: 10.1038/s41579-018-0024-1, PMID: 29789680

[ref3] Bastian M. Heymann S. Jacomy M. (2009). Gephi: an open source software for exploring and manipulating networks. In: *Proceedings of the international AAAI conference on web and social media*. 3, 361–362.

[ref4] Berg G. Smalla K. (2009). Plant species and soil type cooperatively shape the structure and function of microbial communities in the rhizosphere. FEMS Microbiol. Ecol. 68, 1–13. doi: 10.1111/j.1574-6941.2009.00654.x, PMID: 19243436

[ref5] Bledsoe R. B. Goodwillie C. Peralta A. L. (2020). Long-term nutrient enrichment of an oligotroph-dominated wetland increases bacterial diversity in bulk soils and plant rhizospheres. Msphere 5, e00035–e00020. doi: 10.1128/mSphere.00035-20, PMID: 32434837 PMC7380569

[ref6] Broeckling C. D. Broz A. K. Bergelson J. Manter D. K. Vivanco J. M. (2008). Root exudates regulate soil fungal community composition and diversity. Appl. Environ. Microbiol. 74, 738–744. doi: 10.1128/AEM.02188-07, PMID: 18083870 PMC2227741

[ref7] Dixon P. G. Gibson L. J. (2014). The structure and mechanics of Moso bamboo material. J. R. Soc. Interface 11:20140321. doi: 10.1098/rsif.2014.0321, PMID: 25056211 PMC4233722

[ref8] Engelhardt I. C. Welty A. Blazewicz S. J. Bru D. Rouard N. Breuil M. C.. (2018). Depth matters: effects of precipitation regime on soil microbial activity upon rewetting of a plant-soil system. ISME J. 12, 1061–1071. doi: 10.1038/s41396-018-0079-z, PMID: 29476139 PMC5864200

[ref9] Fan K. Weisenhorn P. Gilbert J. A. Shi Y. Bai Y. Chu H. (2018). Soil pH correlates with the co-occurrence and assemblage process of diazotrophic communities in rhizosphere and bulk soils of wheat fields. Soil Biol. Biochem. 121, 185–192. doi: 10.1016/j.soilbio.2018.03.017

[ref10] Fang H. Liu Y. Bai J. Li A. Deng W. Bai T.. (2022). Impact of Moso bamboo (*Phyllostachys edulis*) expansion into japanese cedar plantations on soil fungal and bacterial community compositions. Forests 13:1190. doi: 10.3390/f13081190

[ref11] Fiore-Donno A. M. Human Z. R. Štursová M. Mundra S. Morgado L. Kauserud H.. (2022). Soil compartments (bulk soil, litter, root and rhizosphere) as main drivers of soil protistan communities distribution in forests with different nitrogen deposition. Soil Biol. Biochem. 168:108628. doi: 10.1016/j.soilbio.2022.108628

[ref12] Fox J. Weisberg S. (2018). An R companion to applied regression. America: Sage publications.

[ref13] Gao C. Montoya L. Xu L. Madera M. Hollingsworth J. Purdom E.. (2019). Strong succession in arbuscular mycorrhizal fungal communities. ISME J. 13, 214–226. doi: 10.1038/s41396-018-0264-0, PMID: 30171254 PMC6298956

[ref14] Gdanetz K. Benucci G. M. N. Vande Pol N. Bonito G. (2017). CONSTAX: a tool for improved taxonomic resolution of environmental fungal ITS sequences. BMC Bioinformatics 18, 538–539. doi: 10.1186/s12859-017-1952-x, PMID: 29212440 PMC5719527

[ref15] Gil-Martínez M. López-García Á. Domínguez M. T. Kjøller R. Navarro-Fernández C. M. Rosendahl S.. (2021). Soil fungal diversity and functionality are driven by plant species used in phytoremediation. Soil Biol. Biochem. 153:108102. doi: 10.1016/j.soilbio.2020.108102

[ref16] Gomes N. C. M. Fagbola O. Costa R. Rumjanek N. G. Buchner A. Mendona-Hagler L.. (2003). Dynamics of fungal communities in bulk and maize rhizosphere soil in the tropics. Appl. Environ. Microbiol. 69, 3758–3766. doi: 10.1128/AEM.69.7.3758-3766.2003, PMID: 12839741 PMC165189

[ref17] Guo W. Zhang J. Sui X. Hu X. Lei G. Zhou Y.. (2022). Compartment niche and bamboo variety influence the diversity, composition, network and potential keystone taxa functions of bacterial communities. Rhizosphere 24:100593. doi: 10.1016/j.rhisph.2022.100593

[ref18] Hannula S. De Boer W. Van Veen J. (2010). In situ dynamics of soil fungal communities under different genotypes of potato, including a genetically modified cultivar. Soil Biol. Biochem. 42, 2211–2223. doi: 10.1016/j.soilbio.2010.08.020

[ref19] Harman G. E. Howell C. R. Viterbo A. Chet I. Lorito M. (2004). Trichoderma species—opportunistic, avirulent plant symbionts. Nat. Rev. Microbiol. 2, 43–56. doi: 10.1038/nrmicro797, PMID: 15035008

[ref01] Hough M. McClure A. Bolduc B. Dorrepaal E. Saleska S. Klepac-Ceraj V.. (2020). Biotic and environmental drivers of plant microbiomes across a permafrost thaw gradient. Front. Microbiol. 11:796. doi: 10.3389/fmicb.2020.00796 32499761 PMC7243355

[ref20] Jones D. L. Hodge A. Kuzyakov Y. (2004). Plant and mycorrhizal regulation of rhizodeposition. New Phytol. 163, 459–480. doi: 10.1111/j.1469-8137.2004.01130.x, PMID: 33873745

[ref21] Kong X. Han Z. Tai X. Jin D. Ai S. Zheng X.. (2020). Maize (*Zea mays L*. Sp.) varieties significantly influence bacterial and fungal community in bulk soil, rhizosphere soil and phyllosphere. FEMS Microbiol. Ecol. 96:fiaa020. doi: 10.1093/femsec/fiaa020, PMID: 32016365

[ref22] Kuzyakov Y. Friedel J. K. Stahr K. (2000). Review of mechanisms and quantification of priming effects. Soil Biol. Biochem. 32, 1485–1498. doi: 10.1016/S0038-0717(00)00084-5

[ref02] Langfelder P. Horvath S. (2012). Fast R functions for robust correlations and hierarchical clustering. J. Stat. Softw. 46. PMC3465711 23050260

[ref23] Li Y. He X. Yuan H. Lv G. (2022). Differed growth stage dynamics of root-associated bacterial and fungal community structure associated with halophytic plant Lycium ruthenicum. Microorganisms 10:1644. doi: 10.3390/microorganisms10081644, PMID: 36014066 PMC9414475

[ref24] Li S. Xie D. Ge X. Dong W. Luan J. (2022). Altered diversity and functioning of soil and root-associated microbiomes by an invasive native plant. Plant and Soil 473, 235–249. doi: 10.1007/s11104-022-05338-z

[ref25] Li Y. Yang Y. Zhang H. Wei G. Li Z. (2021). Rhizosphere bacterial and fungal spatial distribution and network pattern of *Astragalus mongholicus* in representative planting sites differ the bulk soil. Appl. Soil Ecol. 168:104114. doi: 10.1016/j.apsoil.2021.104114

[ref26] Li P. Ye S. Liu H. Pan A. Ming F. Tang X. (2018). Cultivation of drought-tolerant and insect-resistant rice affects soil bacterial, but not fungal, abundances and community structures. Front. Microbiol. 9:1390. doi: 10.3389/fmicb.2018.01390, PMID: 30008701 PMC6033987

[ref27] Liu Q. Wang S. Li K. Qiao J. Guo Y. Liu Z.. (2021). Responses of soil bacterial and fungal communities to the long-term monoculture of grapevine. Appl. Microbiol. Biotechnol. 105, 7035–7050. doi: 10.1007/s00253-021-11542-1, PMID: 34477939

[ref28] Liu L. Yan Y. Ding H. Zhao J. Cai Z. Dai C.. (2021). The fungal community outperforms the bacterial community in predicting plant health status. Appl. Microbiol. Biotechnol. 105, 6499–6513. doi: 10.1007/s00253-021-11486-6, PMID: 34415394

[ref29] Liu J. Yao Q. Li Y. Zhang W. Mi G. Chen X.. (2019). Continuous cropping of soybean alters the bulk and rhizospheric soil fungal communities in a Mollisol of northeast PR China. Land Degrad. Dev. 30, 1725–1738. doi: 10.1002/ldr.3378

[ref30] Lozano Y. M. Aguilar-Trigueros C. A. Roy J. Rillig M. C. (2021). Drought induces shifts in soil fungal communities that can be linked to root traits across 24 plant species. New Phytol. 232, 1917–1929. doi: 10.1111/nph.17707, PMID: 34480754

[ref31] Marschner P. Crowley D. Yang C. H. (2004). Development of specific rhizosphere bacterial communities in relation to plant species, nutrition and soil type. Plant and Soil 261, 199–208. doi: 10.1023/B:PLSO.0000035569.80747.c5

[ref32] Mendes L. W. Kuramae E. E. Navarrete A. A. van Veen J. A. Tsai S. M. (2014). Taxonomical and functional microbial community selection in soybean rhizosphere. ISME J. 8, 1577–1587. doi: 10.1038/ismej.2014.17, PMID: 24553468 PMC4817605

[ref33] Mouhamadou B. Puissant J. Personeni E. Desclos-Theveniau M. Kastl E. Schloter M.. (2013). Effects of two grass species on the composition of soil fungal communities. Biol. Fertil. Soils 49, 1131–1139. doi: 10.1007/s00374-013-0810-x

[ref34] Nannipieri P. Ascher J. Ceccherini M. T. Landi L. Pietramellara G. Renella G.. (2007). Microbial diversity and microbial activity in the rhizosphere. Ciencia del Suelo 25, 89–97.

[ref35] Nehls U. Das A. Neb D. (2016). Carbohydrate metabolism in ectomycorrhizal symbiosis. Mol. Mycorrhizal Symb. 10, 161–178. doi: 10.1002/9781118951446.ch10

[ref36] Nguyen N. H. Song Z. Bates S. T. Branco S. Tedersoo L. Menke J.. (2016). FUNGuild: an open annotation tool for parsing fungal community datasets by ecological guild. Fungal Ecol. 20, 241–248. doi: 10.1016/j.funeco.2015.06.006

[ref37] Oksanen J. Blanchet F. G. Friendly M. Kindt R. Legendre P. McGlinn D.., (2020). Vegan: community ecology package. 2.5-7. Available at: http://CRAN.R-project.org/package=vegan

[ref38] Park M. S. Lee J. W. Kim S. H. Park J. H. You Y. H. Lim Y. W. (2020). Penicillium from rhizosphere soil in terrestrial and coastal environments in South Korea. Mycobiology 48, 431–442. doi: 10.1080/12298093.2020.1823611, PMID: 33312010 PMC7717687

[ref39] Perez-Mon C. Stierli B. Plötze M. Frey B. (2022). Fast and persistent responses of alpine permafrost microbial communities to in situ warming. Sci. Total Environ. 807:150720. doi: 10.1016/j.scitotenv.2021.150720, PMID: 34610405

[ref40] Qin D. You C. Lan W. Wang Y. Yu B. Peng Y.. (2022). Microbial assemblages of Schisandraceae plants and the correlations between endophytic species and the accumulation of secondary metabolites. Plant and Soil 1-23. doi: 10.1007/s11104-022-05729-2

[ref41] R Core Team. (2020). R: a language and environment for statistical computing R foundation for statistical computing. Available at: https://www.R-project.org/

[ref42] Ramakrishnan M. Yrjälä K. Vinod K. K. Sharma A. Cho J. Satheesh V.. (2020). Genetics and genomics of moso bamboo (*Phyllostachys edulis*): current status, future challenges, and biotechnological opportunities toward a sustainable bamboo industry. Food Energy Secur. 9:e229. doi: 10.1002/fes3.229

[ref43] Schmidt J. E. Kent A. D. Brisson V. L. Gaudin A. (2019). Agricultural management and plant selection interactively affect rhizosphere microbial community structure and nitrogen cycling. Microbiome 7, 146–118. doi: 10.1186/s40168-019-0756-9, PMID: 31699148 PMC6839119

[ref44] Schöps R. Goldmann K. Herz K. Lentendu G. Schöning I. Bruelheide H.. (2018). Land-use intensity rather than plant functional identity shapes bacterial and fungal rhizosphere communities. Front. Microbiol. 9:2711. doi: 10.3389/fmicb.2018.02711, PMID: 30515138 PMC6255942

[ref45] Semenov M. V. Krasnov G. S. Semenov V. M. van Bruggen A. H. (2020). Long-term fertilization rather than plant species shapes rhizosphere and bulk soil prokaryotic communities in agroecosystems. Appl. Soil Ecol. 154:103641. doi: 10.1016/j.apsoil.2020.103641

[ref46] Söderberg K. H. Olsson P. A. Bååth E. (2002). Structure and activity of the bacterial community in the rhizosphere of different plant species and the effect of arbuscular mycorrhizal colonisation. FEMS Microbiol. Ecol. 40, 223–231. doi: 10.1111/j.1574-6941.2002.tb00955.x, PMID: 19709230

[ref47] Steer J. Harris J. A. (2000). Shifts in the microbial community in rhizosphere and non-rhizosphere soils during the growth of Agrostis stolonifera. Soil Biol. Biochem. 32, 869–878. doi: 10.1016/S0038-0717(99)00219-9

[ref48] Strickland M. S. Rousk J. (2010). Considering fungal: bacterial dominance in soils–methods, controls, and ecosystem implications. Soil Biol. Biochem. 42, 1385–1395. doi: 10.1016/j.soilbio.2010.05.007

[ref49] Su L. Zhang L. Nie D. Kuramae E. E. Shen B. Shen Q. (2020). Bacterial tomato pathogen *Ralstonia solanacearum* invasion modulates rhizosphere compounds and facilitates the cascade effect of fungal pathogen *fusarium solani*. Microorganisms 8:806. doi: 10.3390/microorganisms8060806, PMID: 32471167 PMC7356623

[ref50] Tedersoo L. Sánchez-Ramírez S. Kõljalg U. Bahram M. Döring M. Schigel D.. (2018). High-level classification of the fungi and a tool for evolutionary ecological analyses. Fungal Divers. 90, 135–159. doi: 10.1007/s13225-018-0401-0

[ref51] Urbina H. Breed M. F. Zhao W. Gurrala K. L. Andersson S. G. Ågren J.. (2018). Specificity in *Arabidopsis thaliana* recruitment of root fungal communities from soil and rhizosphere. Fungal Biol. 122, 231–240. doi: 10.1016/j.funbio.2017.12.013, PMID: 29551197

[ref52] Van Der Heijden M. G. Klironomos J. N. Ursic M. Moutoglis P. Streitwolf-Engel R. Boller T.. (1998). Mycorrhizal fungal diversity determines plant biodiversity, ecosystem variability and productivity. Nature 396, 69–72. doi: 10.1038/23932

[ref53] Van Der Heijden M. G. Martin F. M. Selosse M. A. Sanders I. R. (2015). Mycorrhizal ecology and evolution: the past, the present, and the future. New Phytol. 205, 1406–1423. doi: 10.1111/nph.13288, PMID: 25639293

[ref54] Varsadiya M. Urich T. Hugelius G. Bárta J. (2021). Fungi in permafrost-affected soils of the Canadian Arctic: horizon- and site-specific keystone taxa revealed by co-occurrence network. Microorganisms 9:1943. doi: 10.3390/microorganisms9091943, PMID: 34576837 PMC8466989

[ref55] Veach A. M. Morris R. Yip D. Z. Yang Z. K. Engle N. L. Cregger M. A.. (2019). Rhizosphere microbiomes diverge among *Populus trichocarpa* plant-host genotypes and chemotypes, but it depends on soil origin. Microbiome 7, 76–15. doi: 10.1186/s40168-019-0668-8, PMID: 31103040 PMC6525979

[ref56] Vinale F. Sivasithamparam K. Ghisalberti E. L. Marra R. Woo S. L. Lorito M. (2008). Trichoderma–plant–pathogen interactions. Soil Biol. Biochem. 40, 1–10. doi: 10.1016/j.soilbio.2007.07.002

[ref57] Vishwakarma K. Mishra M. Jain S. Singh J. Upadhyay N. Verma R. K.. (2017). Exploring the role of plant-microbe interactions in improving soil structure and function through root exudation: A key to sustainable agriculture. *Plant-microbe interactions in agro-ecological perspectives*. Springer, Singapore. 467–487.

[ref58] Waldrop M. P. Zak D. R. Blackwood C. B. Curtis C. D. Tilman D. (2006). Resource availability controls fungal diversity across a plant diversity gradient. Ecol. Lett. 9, 1127–1135. doi: 10.1111/j.1461-0248.2006.00965.x, PMID: 16972876

[ref59] Wei D. P. Wanasinghe D. N. Hyde K. D. Mortimer P. E. Xu J. Xiao K.. (2019). The genus Simplicillium. MycoKeys 60, 69–92. doi: 10.3897/mycokeys.60.38040, PMID: 31798310 PMC6879665

[ref60] Ye F. Wang X. Wang Y. Wu S. Wu J. Hong Y. (2021). Different pioneer plant species have similar rhizosphere microbial communities. Plant and Soil 464, 165–181. doi: 10.1007/s11104-021-04952-7

[ref61] Zhang K. Adams J. M. Shi Y. Yang T. Sun R. He D.. (2017). Environment and geographic distance differ in relative importance for determining fungal community of rhizosphere and bulk soil. Environ. Microbiol. 19, 3649–3659. doi: 10.1111/1462-2920.13865, PMID: 28752936

[ref62] Zheng H. Yang T. Bao Y. He P. Yang K. Mei X.. (2021). Network analysis and subsequent culturing reveal keystone taxa involved in microbial litter decomposition dynamics. Soil Biol. Biochem. 157:108230. doi: 10.1016/j.soilbio.2021.108230

