# Supplementary material for: Soil fungal community characteristics vary with bamboo varieties and soil compartments
Source: Front Microbiol. 2023 Feb 6;14:1120679. doi: 10.3389/fmicb.2023.1120679 (PMC9939831; doi:10.3389/fmicb.2023.1120679)
Supplement: Supplementary file 1 [file Data_Sheet_1.docx]

**Soil fungal community characteristics vary with both bamboo variety and soil compartment**

**Wen Guo ^1, 2^, Jian Zhang ^1^, Mai-He Li ^2,3,4*^, Lianghua Qi ^1,5*^**

**Supplemental Material**

**Table S1.** Fungal community differences among the soil compartments and bamboo varieties. Results of the permutation multivariate analysis of variance (PERMANOVA) are shown.

| **Comparison** | **PERMANOVA** | |
| --- | --- | --- |
|  | **Variation (R^2^)** | ***P*** |
| **All** | 0.815 | 0.001 |
| **Rhizosphere & Bulk soil** | 0.138 | 0.013 |
| **Bulk soil** | 0.488 | 0.001 |
| **Rhizosphere** | 0.441 | 0.004 |

**Table S2.** Relative abundances (mean ± SE) of dominant fungi (phylum level) in the different soil compartments (rhizosphere and bulk soil) and under different bamboo varieties.

Soil compartment

| **Soil compartment** | **Ascomycota** | **Basidiomycota** | **Glomeromycota** | **Mortierellomycota** | **Mucoromycota** | **Chytridiomycota** | **other** |
| --- | --- | --- | --- | --- | --- | --- | --- |
| **Bulk soil** | 36.28±3.52a | **30.37±4.98a** | **0.63±0.11a** | 17.90±4.68a | 13.59±2.17a | **0.91±0.13a** | 0.32±0.15a |
| **Rhizosphere** | 44.06±3.24a | **16.09±3.27b** | **0.28±0.10b** | 23.24±4.31a | 16.14±2.83a | **0.08±0.03b** | 0.11±0.07a |

Bamboo variety

| **Variety** | **Soil compartment** | **Ascomycota** | **Basidiomycota** | **Glomeromycota** | **Mortierellomycota** | **Mucoromycota** | **Chytridiomycota** | **other** |
| --- | --- | --- | --- | --- | --- | --- | --- | --- |
| **PE** | Bulk soil | 45.60±3.80a | 25.77±5.03a | 0.85±0.21a | 7.75±0.92b | 18.66±7.07a | 1.31±0.20a | 0.05±0.00b |
| **FT** |  | 41.87±5.95a | 21.18±4.89a | 0.68±0.33a | 16.17±6.05b | 19.02±2.93a | 0.86±0.20a | 0.22±0.10b |
| **FL** |  | 38.57±6.95a | 40.37±6.05a | 0.73±0.38a | 10.80±2.31b | 8.89±1.57a | 0.61±0.32a | 0.04±0.02b |
| **FP** |  | 21.74±4.59a | 15.58±4.13a | 0.41±0.03a | 4**5.69±14.33a** | 14.19±5.85a | 1.21±0.34a | 1.20±0.57a |
| **FG** |  | 33.63±12.03a | 48.96±19.49a | 0.46±0.24a | 9.09±3.78b | 7.20±3.17a | 0.57±0.32a | 0.10±0.05b |
| **PE** | Rhizosphere | 53.99±8.39a | **34.13±3.71a** | **0.99±0.14a** | **1.82±0.45b** | 8.79±3.62a | 0.13±0.05ab | 0.16±0.06a |
| **FT** |  | 41.28±3.46a | 15.83±4.68b | 0.04±0.01b | 24.42±5.13ab | 18.42±3.02a | **0.01±0.00b** | 0.00±0.00a |
| **FL** |  | 38.38±2.17a | 12.57±4.52b | 0.11±0.06b | 26.26±4.80ab | 22.62±8.13a | **0.04±0.02b** | 0.02±0.01a |
| **FP** |  | 35.67±6.36a | 15.52±7.48b | 0.21±0.10b | 28.39±10.84ab | 19.62±9.99a | **0.23±0.10a** | 0.36±0.15a |
| **FG** |  | 50.95±10.55a | 2.39±0.52b | 0.07±0.03b | **35.33±12.79a** | 11.25±2.46a | **0.00±0.00b** | 0.00±0.00a |

**Note****:** **PE** *Phyllostachys edulis*; **FT** *P. edulis* f. *tao kiang*; **FL** *P. edulis* f. *luteosulcata*; **FP** *P. edulis* f. *pachyloen*; **FG** *P. edulis* f. *gracilis*. Different lowercase letters indicate that there are significant differences in the relative abundance of the fungi among the different soil compartments/bamboo varieties at the same phylum level (*P* < 0.05). The part in bold indicates a higher value.

**Table S3.** Relative abundances (mean ± SE) of dominant soil fungi (genus level) in the different soil compartments (rhizosphere and bulk soil) and under different bamboo varieties.

Soil compartment

| **Soil compartment** | **Umbelopsis** | **Trichoderma** | **Mortierella** | **Saitozyma** | **Penicillium** | **Talaromyces** | **other** |
| --- | --- | --- | --- | --- | --- | --- | --- |
| **Bulk soil** | 13.74±2.92a | 12.05±1.94a | 23.05±4.97a | 8.78±2.09a | 9.22±2.46a | 1.66±0.28a | 31.51±3.80a |
| **Rhizosphere** | 12.28±2.22a | 11.82±1.99a | 25.75±4.67a | 10.71±3.35a | 14.02±3.42a | 1.70±0.32a | 23.72±3.78a |

Bamboo variety

| **Variety** | **Soil compartment** | **Umbelopsis** | **Trichoderma** | **Mortierella** | **Saitozyma** | **Penicillium** | **Talaromyces** | **other** |
| --- | --- | --- | --- | --- | --- | --- | --- | --- |
| **PE** | Bulk soil | 23.74±9.41a | 12.79±2.20b | 10.75±1.45b | 20.28±5.65a | 4.27±0.03b | 2.13±0.54a | 26.03±2.92b |
| **FT** |  | 12.97±5.45a | 7.35±2.59b | 20.45±7.13b | 8.23±1.64b | 12.91±3.93ab | 2.01±0.31a | 36.08±5.34ab |
| **FL** |  | 8.26±2.56a | 9.32±2.50b | 16.51±3.36b | 5.47±0.50b | 7.67±2.77ab | 1.25±0.32a | 51.52±3.11a |
| **FP** |  | 16.37±8.40a | 6.56±1.48b | 53.01±14.57a | 3.02±1.97b | 2.63±0.84b | 0.73±0.35a | 17.69±3.64b |
| **FG** |  | 7.36±3.73a | 24.21±4.66a | 14.53±1.89b | 6.89±2.89b | 18.63±9.96a | 2.17±1.09a | 26.22±10.44b |
| **PE** | Rhizosphere | 5.11±1.88a | 16.01±3.85a | 2.83±0.64b | 33.65±3.14a | 5.03±0.49a | 1.20±0.27ab | 36.18±3.27a |
| **FT** |  | 14.65±2.6a | 9.16±1.43a | 26.13±5.31ab | 13.00±2.84b | 13.32±2.20a | 2.94±1.10a | 20.80±5.24ab |
| **FL** |  | 13.16±3.98a | 15.61±3.84a | 30.14±6.23ab | 3.77±0.93c | 9.03±1.90a | 1.27±0.47ab | 27.04±3.33ab |
| **FP** |  | 17.76±9.78a | 5.61±2.11a | 33.03±12.87ab | 2.50±0.43c | 13.87±5.62a | 0.73±0.34b | 26.51±10.06ab |
| **FG** |  | 10.75±2.25a | 12.72±7.94a | 36.62±13.16a | 0.61±0.03c | 28.88±14.32a | 2.34±0.56ab | 8.07±3.13b |

**Note:** **PE** *Phyllostachys edulis*; **FT** *P. edulis* f. *tao kiang*; **FL** *P. edulis* f. *luteosulcata*; **FP** *P. edulis* f. *pachyloen*; **FG** *P. edulis* f. *gracilis*. Different lowercase letters indicate that there are significant differences in the relative abundance of the fungi among different soil compartments/bamboo varieties at the same genus level (*P* < 0.05).

**Table S4.** Effects of soil compartment and bamboo variety on the relative abundance of dominant fungal taxa at the phylum and the genus level, based on a two-way analysis of variance (ANOVA; F and P values are listed).

|  | **Taxonomy** |  | **Compartment (C)** | **Variety**  **(V)** | **C × V** |
| --- | --- | --- | --- | --- | --- |
| **Phylum** | Ascomycota | *F* | 0.87 | 8.51 | 1.63 |
|  |  | *P* | 0.35 | 0.07 | 0.8 |
|  | Basidiomycota | *F* | 5.3 | 4.92 | 10.44 |
|  |  | *P* | **<0.05** | 0.30 | **<0.05** |
|  | Mucoromycota | *F* | 6.2 | 8.96 | 2.94 |
|  |  | *P* | **<0.05** | 0.06 | 0.56 |
|  | Mortierellomycota | *F* | 2.04 | 13.73 | 6.41 |
|  |  | *P* | 0.15 | **<0.01** | 0.17 |
|  | Chytridiomycota | *F* | 0.31 | 4.05 | 5.55 |
|  |  | *P* | 0.58 | 0.40 | 0.24 |
|  | Glomeromycota | *F* | 14.92 | 6.34 | 0.49 |
|  |  | *P* | **<0.01** | 0.18 | 0.97 |
| **Genus** | Umbelopsis | *F* | 0.1 | 1.72 | 4.81 |
|  |  | *P* | 0.76 | 0.79 | 0.31 |
|  | Trichoderma | *F* | 0.03 | 10.77 | 4.92 |
|  |  | *P* | 0.85 | **<0.05** | 0.3 |
|  | Mortierella | *F* | 0.53 | 14.09 | 4.66 |
|  |  | *P* | 0.46 | **<0.01** | 0.32 |
|  | Saitozyma | *F* | 0.16 | 21.01 | 2.29 |
|  |  | *P* | 0.69 | **<0.01** | 0.68 |
|  | Penicillium | *F* | 2.68 | 13.03 | 2.16 |
|  |  | *P* | 0.1 | **<0.05** | 0.71 |
|  | Talaromyces | *F* | 0.07 | 7.8 | 1.28 |
|  |  | *P* | 0.79 | 0.09 | 0.87 |
|  | Gongronella | *F* | 0.76 | 19.48 | 0.75 |
|  |  | *P* | 0.38 | **<0.01** | 0.94 |
|  | Mycena | *F* | 2.29 | 14.93 | 4.2 |
|  |  | *P* | 0.13 | **<0.01** | 0.38 |
|  | Trechispora | *F* | 7.86 | 2.56 | 3.65 |
|  |  | *P* | **<0.01** | 0.63 | 0.46 |
|  | Arcopilus | *F* | 0.36 | 20.8 | 0.16 |
|  |  | *P* | 0.55 | **<0.01** | 0.99 |

**Table S5.** Properties of the fungal co-occurrence networks in the two soil compartments (rhizosphere and bulk soil).

| **Soil compartment** | **Number of nodes** | **Number of edges** | **Average degree** | **Network diameter** | **Clustering coefficient** | **Average path length** | **Positive/**  **Negative (%)** |
| --- | --- | --- | --- | --- | --- | --- | --- |
| **Bulk soil** | 326 | 2304 | 14.135 | 16 | 0.761 | 5.878 | 99.96/0.04 |
| **Rhizosphere** | 262 | 2710 | 20.687 | 8 | 0.821 | 3.478 | 100/0 |

**Table S6.** Taxonomy of the two potential keystone taxa of the fungal networks in the rhizosphere and in the bulk soil.

| **Soil compartment** | **Phylum** | **Class** | **Order** | **Family** | **Genus** |
| --- | --- | --- | --- | --- | --- |
| **Bulk soil** | Ascomycota | Sordariomycetes | Hypocreales | Cordycipitaceae | Simplicillium |
|  | Ascomycota | Sordariomycetes | Hypocreales | Nectriaceae | Nectria |
| **Rhizosphere** | Ascomycota | Eurotiomycetes | Eurotiales | Aspergillaceae | Penicillium |
|  | Basidiomycota | Agaricomycetes | Corticiales | Corticiaceae | Laetisaria |

**Table S7.** Two-way analysis of variance (ANOVA; *F* and *P* values are shown) comparing the fungal trophic modes in the different soil compartments (rhizosphere and bulk soil) and under the different bamboo varieties. Significant differences (*P* < 0.05) are given in bold.

| **Trophic modes** |  | **Compartment (C)** | **Variety (V)** | **C × V** |
| --- | --- | --- | --- | --- |
| Pathogen-Saprotroph-Symbiotroph | *F* | 6.51 | 6.57 | 1.68 |
|  | *P* | **<0.05** | 0.16 | 0.79 |
| Pathotroph | *F* | 8.55 | 7.78 | 1.45 |
|  | *P* | **<0.01** | 0.10 | 0.84 |
| Pathotroph-Saprotroph | *F* | 2.68 | 14.00 | 7.93 |
|  | *P* | 0.10 | **<0.01** | 0.09 |
| Pathotroph-Saprotroph-Symbiotroph | *F* | 0.23 | 20.14 | 2.08 |
|  | *P* | 0.63 | **<0.01** | 0.72 |
| Pathotroph-Symbiotroph | *F* | 13.33 | 8.26 | 1.83 |
|  | *P* | **<0.01** | 0.08 | 0.76 |
| Saprotroph | *F* | 0.01 | 2.19 | 1.60 |
|  | *P* | 0.94 | 0.11 | 0.21 |
| Saprotroph-Symbiotroph | *F* | 0.27 | 6.11 | 5.08 |
|  | *P* | 0.60 | 0.19 | 0.28 |
| Symbiotroph | *F* | 4.39 | 3.94 | 4.76 |
|  | *P* | **<0.05** | 0.41 | 0.31 |

**Table S8.** Two-way analysis of variance (ANOVA; *F* and *P* values are shown) comparing the fungal functional guilds in the different soil compartments (rhizosphere and bulk soil) and under the different bamboo varieties. Significant differences (*P* < 0.05) are given in bold.

| **Functional guild** |  | **Compartment (C)** | **Variety (V)** | **C × V** |
| --- | --- | --- | --- | --- |
| Wood saprotroph | *F* | 0.23 | 20.84 | 1.25 |
|  | *P* | 0.63 | **<0.01** | 0.87 |
| Soil saprotroph | *F* | 4.41 | 9.35 | 3.70 |
|  | *P* | **<0.05** | 0.06 | 0.45 |
| Plant saprotroph | *F* | 0.94 | 9.89 | 2.65 |
|  | *P* | 0.33 | **<0.05** | 0.62 |
| Leaf saprotroph | *F* | 0.01 | 3.11 | 5.18 |
|  | *P* | 0.96 | 0.54 | 0.27 |
| Undefined saprotroph | *F* | 3.26 | 10.05 | 4.41 |
|  | *P* | 0.07 | **<0.05** | 0.35 |
| Animal pathogen | *F* | 12.60 | 10.64 | 2.63 |
|  | *P* | **<0.01** | **<0.05** | 0.62 |
| Plant pathogen | *F* | 9.04 | 5.84 | 0.64 |
|  | *P* | **<0.05** | 0.21 | 0.96 |
| Lichen parasite | *F* | 0.46 | 5.96 | 8.72 |
|  | *P* | 0.50 | 0.20 | 0.07 |
| Fungal parasite | *F* | 11.57 | 11.59 | 2.65 |
|  | *P* | **<0.01** | **<0.05** | 0.62 |
| Lichenized | *F* | 0.36 | 12.85 | 1.43 |
|  | *P* | 0.55 | **<0.05** | 0.84 |
| Epiphyte | *F* | 3.42 | 9.04 | 1.79 |
|  | *P* | 0.06 | 0.06 | 0.77 |
| Endophyte | *F* | 1.40 | 11.64 | 3.19 |
|  | *P* | 0.24 | **<0.05** | 0.53 |
| Ectomycorrhizal | *F* | 4.80 | 8.07 | 1.09 |
|  | *P* | **<0.05** | 0.09 | 0.90 |
| Orchid mycorrhizal | *F* | 1.58 | 7.14 | 1.51 |
|  | *P* | 0.21 | 0.13 | 0.82 |
| Arbuscular mycorrhizal | *F* | 7.51 | 11.74 | 2.16 |
|  | *P* | **<0.01** | **<0.05** | 0.71 |

**Figure S1** Prediction of the fungal functional guilds (trophic modes) based on the FUNGuild database.

**
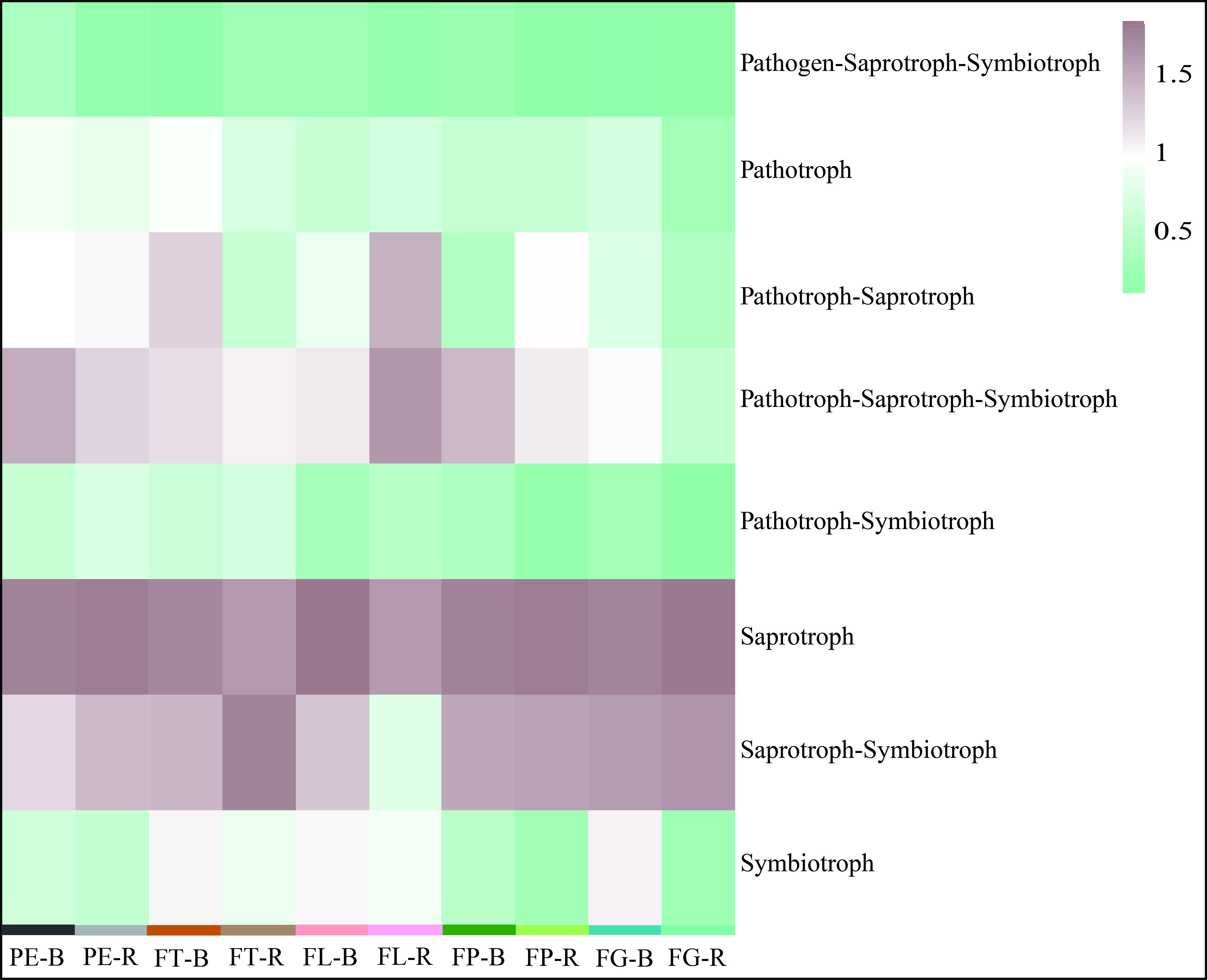
**
